# Lipoprotein(a): a potential target for the reduction of residual cardiovascular risk in patients with chronic kidney disease

**DOI:** 10.3389/fneph.2026.1950030

**Published:** 2026-10-07

**Authors:** Leonardo A. Sechi, Luca Bulfone, Antonio Vacca, Jacopo Comand, Stefano Marcante, Gabriele Brosolo, Claudio Pagano, Cristiana Catena

**Affiliations:** Clinica Medica, Department of Medicine, University of Udine, Udine, Italy

**Keywords:** antisense oligonucleotides, cardiovascular events, chronic kidney disease, glomerular filtration rate, lipoprotein(a), muvalaplin, residual cardiovascular risk, small interfering RNA

## Abstract

Epidemiological evidence demonstrates that atherosclerotic cardiovascular disease (ASCVD) is a major cause of death and disability in patients with chronic kidney disease (CKD). From the early stages of CKD, and in addition to specifically related conditions, the coexistence of traditional risk factors contributes to a process of accelerated atherosclerosis. In patients with CKD, increased cardiovascular risk persists even after correction of hypertension, dyslipidemia, and diabetes, indicating a residual risk likely related to additional subtle factors. Lipoprotein(a) [Lp(a)] is an LDL-like particle containing apoB100 covalently linked to apo(a) and is now recognized as a genetically determined risk factor for ASCVD. Following its initial description, Lp(a) aroused extraordinary interest, which then waned in the late-1990s due to the controversial results of some prospective studies and the lack of effective interventions to reduce its circulating levels. Over the past two decades, clinical studies have provided robust evidence supporting the causal and independent contribution of Lp(a) to ASCVD, also suggesting a significant contribution to residual cardiovascular risk. This contribution may be particularly significant in patients with CKD, who exhibit elevated Lp(a) levels from the earliest stages of disease. The recent development of potent Lp(a)-lowering drugs offers an exciting opportunity to effectively address residual risk in the general population and in patients with CKD. No data are yet available on their use in patients with CKD but pending future definition of their efficacy on cardiovascular outcomes, approval, dosing, and long-term safety, these drugs might possibly represent a useful tool for cardiovascular prevention in these patients.

## 1 Introduction

After the discovery of lipoprotein(a) [Lp(a)], observational studies conducted in the late 1980s reported a significant association of its plasma levels with coronary heart disease (1). Since the results of the first prospective studies were discordant on the role of Lp(a) as a risk factor for atherosclerotic cardiovascular disease (ASCVD) (2) and did not provide clear evidence of a causal role, this lipoprotein was initially considered only as a disease marker. For this reason, its scientific interest was dampened for a couple of decades, also due to the lack of effective dietary or pharmacologic interventions for the reduction of its circulating levels. Over the past two decades, however, a long list of well conducted investigations (population-based studies; meta-analyses of prospective studies; genome-wide association studies; Mendelian randomization studies) (3–5) has completely changed the picture, and Lp(a) has triggered renewed interest, thus emerging as a strong independent and genetically determined causal risk factor for ASCVD (6, 7). Indeed, plasma Lp(a) levels have been used to redefine the cardiovascular risk category in approximately 40% of primary prevention patients classified as intermediate risk according to the Framingham risk score (8). Furthermore, results from recent primary and secondary prevention studies underline the evidence that elevated Lp(a) levels amplify the risk of events despite optimal control of additional risk factors (9, 10). Thus, strong evidence currently supports the hypothesis that elevated circulating Lp(a) levels are a key factor in “residual cardiovascular risk” and, in line with currently available data, many recent guidelines and consensus statements on cardiovascular prevention recommend widespread testing for Lp(a) (11).

Epidemiological evidence clearly indicates that cardiovascular events are the main cause of disability and mortality in patients with chronic kidney disease (CKD) (12). The mechanisms involved include both conditions specific to kidney disease (anemia, electrolyte and acid-base disturbances, uremia, etc.) and classical cardiovascular risk factors (diabetes, hypertension, dyslipidemia). If identified early, all these conditions can be effectively corrected in an attempt to reduce the cardiovascular risk of patients with progressive deterioration of kidney function. Other conditions, however, commonly identified as “emerging” risk factors, have been implicated as significant contributors to the process of accelerated atherosclerosis typically observed in these patients (13). Many of these factors may impact cardiovascular risk from the early stages of CKD (14–17) and account for a significant portion of the residual cardiovascular risk that occurs in these patients and is not modified by the correction of traditional risk factors. Among these emerging factors, Lp(a) may play a prominent role as its plasma levels gradually increase while renal function progressively worsens (17–20). Furthermore, elevated Lp(a) levels are independently associated with evidence of increased cardiovascular damage (21–23).

As previously highlighted, currently available dietary and lipid-lowering treatments have shown only modest and clinically irrelevant effects on circulating Lp(a) levels, and therefore, evidence that correcting these levels improves cardiovascular outcomes is lacking. In this context, however, the recent development of effective Lp(a)-lowering drugs has laid the foundation for testing the hypothesis that Lp(a) reduction actually affects the residual risk of atherosclerotic cardiovascular disease, an effect that could be particularly relevant in patients with impaired kidney function. The aim of this article is to examine the possible impact of Lp(a) to the residual cardiovascular risk of patients with CKD, from the early stages of the disease. We also overview the potential future benefits that might be obtained with the use, in these patients, of drugs that have the capacity to reduce Lp(a) levels. For the purpose of this review, we performed a PubMed search for English-language articles published up to July 2026, using “lipoprotein(a)”, “residual cardiovascular risk”, and “kidney disease” as Medical Subject Headings and title keywords. Clinical studies and comprehensive reviews were considered, while animal studies, editorials, and case reports were excluded. Articles were independently screened by two authors (LAS and CC) and subsequently selected during preparation of the first draft of the article.

## 2 Lipoprotein(a): structure, genetics and metabolism

Lp(a) is a low-density lipoprotein (LDL) in which apolipoprotein B is covalently linked, in a one-to-one molar ratio, to apolipoprotein(a) [apo(a)], a glycoprotein of variable molecular weight (24). The lipid portion of Lp(a) is the same as LDL, containing triglycerides and esterified cholesterol surrounded by a layer of non-esterified cholesterol, phospholipids, and proteins. The size variability of apo(a) partially accounts for the plasma Lp(a) level, with smaller apo(a) sizes being associated with higher plasma Lp(a) concentrations. The apo(a) gene (LPA) is located on chromosome 6 and encodes a protein that shares important structural homology with plasminogen (25). Both apo(a) and plasminogen contain multiple triple-ring structures called kringles. Apo(a) consists of multiple repeated copies of sequences homologous to the kringle-4 (K4) domain of plasminogen, followed by a single kringle-5-like domain and an inactive protease-like domain. Apo(a) contains 10 subtypes of K4 repeats (K4 type-1 to K4 type-10) that differ from each other based on their amino acid sequence. All K4 kringle types are present in a single copy within the Lp(a) moiety, with the notable exception of K4-2 type, which is present in a variable number of identically repeated copies, usually from 3 to more than 40, which are encoded by the LPA gene (24). The K4-2 copy number is highly variable across individuals and ancestry groups explaining part of the interindividual Lp(a) levels variability, while other genetic variants of LPA and, to a lesser extent, loci outside LPA also contribute.

The LPA gene is highly expressed in the liver, where it is linked to the plasminogen gene, thereby explaining the strong structural homology between the two (over 80%). The LPA gene comprises several regions, including the sequence encoding the plasminogen-like domain with multiple tandem repeats (K4), the sequence encoding the signal peptide, and the sequence encoding a plasminogen-like protease domain (25). The extensive size heterogeneity of LPA results in a highly variable apo(a) size polymorphism, which is the major determinant of Lp(a) size and causes substantial variation in molecular mass and interindividual differences. Homozygosity for apo(a) size is rare, and most individuals in the general population express two different sizes of apo(a) isoforms (26). As previously stated, there is an inverse association between the number of K4-2 repeats and plasma Lp(a) levels (26). This inverse association might be related to slower cellular assembly and slower secretion rate of larger Lp(a) molecules (27).

Regulation of LPA expression occurs at both the transcriptional and post-transcriptional levels, and the regulatory factors involved are under active investigation. A role for acute phase reactants and sex hormones has been suggested in human studies (28), while studies on LPA regulation in transgenic mice expressing apo(a) have identified two gene enhancer sites that could be activated by nuclear transcription factors (29). Further studies conducted in established liver cell lines (HepG2) have identified additional regulatory regions with opposing influences on LPA expression (30). Endogenous and exogenous triggers have been shown to modulate the function of these regions, although the regulatory mechanisms of LPA remain uncertain and merit further investigation. Importantly, circulating Lp(a) levels are influenced by additional genetic determinants other than LPA. These determinants include several single nucleotide polymorphisms (SNPs) within the structural and functional domains, some variants affecting RNA splicing, and pentanucleotide repeats located in the promoter region (6). Approximately 200 SNPs have been identified by genome-wide association studies, reporting different influence on circulating Lp(a) levels. Some of these SNPs are associated with high Lp(a) levels, while others are associated with low Lp(a) levels. However, not all variants have been shown to be relevant in predicting Lp(a) levels (6).

The entire process of Lp(a) production, from LPA transcription to the final assembly of the protein into the Lp(a) molecule, occurs in the liver. Hepatocytes secrete lipoproteins containing apo(a) and apoB separately, and the final assembly of Lp(a) particles occurs outside the cell by covalent linkage of apoB with apo(a). Synthesis and secretion are regulated by the effects of genetic control of LPA expression and processing of the apo(a) protein, respectively. Recognition of the importance of these steps in Lp(a) production has laid the foundation for the development of agents that interfere with LPA expression (antisense oligonucleotides; small interfering RNAs) or apolipoprotein assembly (small-molecule oral inhibitors), thereby effectively reducing circulating Lp(a) levels.

Similar to biosynthesis, the catabolic pathways of Lp(a) are not entirely clear. The involvement of endocytic cellular receptors, LDL receptors regulated by the activity of proprotein convertase subtilisin/kexin type 9 (PCSK9), LDL receptor-related protein 1, the very low-density lipoprotein receptor, and the plasminogen activator (KT) receptor, has been hypothesized (31). In this context, while most receptors deliver the entire Lp(a) molecule to lysosomal degradation, the plasminogen activator (KT) receptor recycles apo(a) into a new Lp(a) molecule. Furthermore, and importantly, the kidney appears to play a major role in the clearance of Lp(a) from the circulation. Clinical studies have clearly indicated that plasma Lp(a) increases progressively as glomerular filtration rate (GFR) decreases (17, 18, 20, 32).

In summary, there is evidence strongly suggesting that the kidney participates in the catabolism of Lp(a) or apo(a), although the specific metabolic pathways and the relative importance of reduced renal clearance versus increased hepatic production need to be further investigated.

Reproducible and reliable measurement of Lp(a) has proven difficult to achieve primarily due to the high polymorphism of the apo(a) moiety, resulting from the variability in isoform size. Additional factors included lack of standardization among laboratories with some tests reporting Lp(a) values as mass concentrations (mg/dL) and others as particle concentrations (nmol/L), and the adoption of antibody-based approaches that could lead to possible underestimation of smaller isoforms and overestimation of larger isoforms. A conversion factor between mg/dL and nmol/L has come into use but is entirely inappropriate since the size of the apo(a) isoforms modifies the mass of the Lp(a) particle. In fact, even the risk thresholds expressed in mg/dL and nmol/L identify approximate clinical cut-off values rather than exact equivalents. For this reason, efforts to standardize reference material for Lp(a) assay calibration have aimed to improve reproducibility between methods, although some heterogeneity persists and use of apo(a)-isoform-insensitive assays should be broadly recommended (33). The recently developed LC-MS/MS method, based on selected proteotypic peptides quantified by isotope dilution, is independent of isoforms, overcoming some of the limitations and allowing better assay standardization. Reports with multiple serial measurements of Lp(a) indicate that temporal variability of plasma levels is on average rather modest (33). A study of 145 patients with an average of 6 measurements over 1.7 years showed a coefficient of variation of 18% (34). The extent of this variability might lead to conclude that for most subjects a single measurement of Lp(a) may be sufficient for a risk estimation. However, Lp(a) fluctuations might be relevant to patients who are included in Lp(a)-intervention trials. Although plasma Lp(a) levels are not significantly affected by dietary or other lifestyle changes, significant changes of Lp(a) resulting from pathological conditions such as acute inflammatory states and progressive renal diseases undergoing various types of treatment must be taken into account (35). These aspects could motivate the performance of repeated measurements of Lp(a).

## 3 The problem of residual cardiovascular risk

Residual cardiovascular risk identifies the probability of experiencing a major adverse cardiovascular event despite treatment of traditional risk factors (blood pressure, LDL cholesterol, blood glucose, obesity, smoking) according to best standards of care. Contributors to residual cardiovascular risk, including lipid, metabolic, thrombotic, and inflammatory pathways, together with a long list of emerging factors, have a pivotal role beyond traditional determinants of cardiovascular risk (36). Residual cardiovascular risk is important at the epidemiological level for implementing more realistic predictive models and at the public health level for developing new treatment guidelines. New biomarkers and mechanistic insights can improve patient stratification and guide targeted treatments aimed at reducing recurrent events.

Epidemiological evidence clearly demonstrates that effective blood pressure control in hypertensive subjects is associated with a significant reduction in all cardiovascular and renal complications. However, although the appropriate use of antihypertensive drugs normalizes blood pressure levels, it cannot completely reverse the risk of hypertension-related morbid events. In the PRIME (Prospective Epidemiological Study of Myocardial Infarction) study, 9649 men aged 50-59 years were followed for 10 years to investigate the presence of residual cardiovascular risk in those who had been treated with antihypertensive drugs (37). In this cohort, 796 patients had a major cardiovascular event and, after adjustment for concurrent risk factors, the use of antihypertensive drugs at the time of recruitment was associated with a relative risk of events increased by 50%. In the Framingham Offspring Cohort study, 3024 participants were assigned to different categories based on systolic and diastolic blood pressure levels and the use of antihypertensive agents (38). After statistical adjustments, treated hypertensive patients with blood pressure <140/90 mm Hg or blood pressure ≥ 140/90 had a 50% and 28% higher risk of cardiovascular events, respectively, compared to untreated subjects with comparable blood pressure levels. Similar observations have been reported in other studies highlighting the relevance of residual risk in hypertension despite effective blood pressure reduction (39).

Among the factors that may contribute to residual risk in hypertensive patients effectively treated, a significant role may be attributed to a greater burden of subclinical cardiac, vascular and renal damage (38). Genetic factors may also contribute to residual risk (40), along with a long list of emerging risk factor not controlled by antihypertensive treatment. Among these emerging factors, Lp(a) may play a prominent role. In 277 untreated patients with mild-to-moderate hypertension, we measured plasma Lp(a) and characterized apo(a) phenotypes based on their molecular weight (21). Although plasma Lp(a) levels were comparable in hypertensive patients and matched controls, its levels were progressively and significantly higher at different stages of hypertension-related subclinical and clinical organ damage. Furthermore, higher Lp(a) levels and greater organ damage were associated with a higher frequency of low molecular weight apo(a) isoforms, strongly suggesting a genetic predisposition to Lp(a)-related hypertensive complications.

Another critical component of ASCVD reduction is achieving plasma LDL targets. Despite the availability of multiple, cost-effective medications and significantly greater achievement of LDL targets, the higher risk of ASCVD persists compared with subjects with plasma lipids within normal limits (41). A long-term reassessment of early large-scale studies of lipid-lowering treatments have demonstrated that more intensive LDL reduction does not necessarily eliminate ASCVD-related mortality, and studies with follow-up longer than 1 year have reported a residual risk of approximately 30% (42). More recent findings from the Treating to New Targets (TNT) (43), Incremental Decrease in End Points Through Aggressive Lipid Lowering (IDEAL) (44), and Collaborative Atorvastatin Diabetes Study (CARDS) (45) studies revealed that a large number of patients experienced substantial residual event rates despite meaningful LDL reduction. Even trials with the most potent PCSK9-inhibitors, in which circulating LDL levels were reduced to a median of less than 30 mg/dL, demonstrated that even extreme LDL reduction was not sufficient to normalize cardiovascular risk (46, 47). The persistence of increased ASCVD incidence despite maximal LDL reduction has sparked increased interest in identifying potential drivers of residual risk, and Lp(a) has emerged as a major contributor to this risk (41). Most studies of lipid-lowering drugs, including those with the most potent PCSK9-inhibitors, have reported modest and clinically irrelevant effects on circulating Lp(a). A recent participant-level meta-analysis demonstrated that effective LDL reduction does not offset Lp(a)-related ASCVD risk, highlighting the additive and independent contribution of Lp(a) to this risk (48). Although renal function was not included as a covariate in this study (49), participants with Lp(a) >50 mg/dL and LDL <77 mg/dL still had a 38% greater risk compared with patients with lower plasma Lp(a) levels. For this reason, Lp(a) might represent a primary target for further interventions using newer, specifically targeted, drug treatments, currently in various stages of development.

Although circulating Lp(a) levels increase the risk of atherosclerotic and thrombotic complications along a continuum, values above 50 mg/dL (>125 nmol/L) are conventionally considered elevated, and likely to sharply increase the risk. Approximately 15-20% of people worldwide have Lp(a) levels above 50 mg/dL. The distribution of Lp(a) is strongly skewed towards lower values, ranging from less than 0.2 to more than 200 mg/dL. Although plasma Lp(a) level is largely stable, this can fluctuate slightly increasing by 10-20% due to inflammatory conditions, postmenopausal status, and use of some drugs (e.g. statins) (35). There is considerable variability in median Lp(a) levels across ethnic groups (Asians generally have lower levels; Africans have higher levels with larger isoforms; Caucasians exhibit greater variability, with a higher prevalence of small isoforms), but despite this variability, elevated Lp(a) levels are associated with a comparable increase of relative risk of ASCVD across ethnic groups (50).

## 4 Cardiovascular risk in patients with chronic kidney disease and the role of Lp(a)

Strong epidemiological evidence clearly demonstrates that patients with impaired kidney function are at increased risk for cardiovascular events. An association between ASCVD and CKD starting from the early stages of disease was initially suggested in sub-analyses of large epidemiological studies in patients with hypertension (51). The stepwise association between progressive decline in eGFR (estimated GFR) and ASCVD was conclusively demonstrated in two pivotal studies published in 2004. In the VALIANT (Valsartan in Acute Myocardial Infarction Trial) study, which enrolled patients with myocardial infarction and baseline eGFR <81 mL/min/1.73 m2, the relative risk of ASCVD increased by 10% for every 10 mL/min/1.73 m2 reduction in eGFR (52). In a prospective study conducted at Kaiser Permanente, over one million adults were enrolled in a 2.8-year follow-up program, which showed a progressive increase in cardiovascular events as baseline eGFR fell below 60 ml/min/1.73 m2 (53). Further evidence has accumulated in subsequent studies, clearly demonstrating that even mild impairment of kidney function significantly increases the risk of ASCVD (54).

It is important to keep in mind that, in patients with CKD, the burden of cardiovascular disease is not limited to ASCVD. Indeed, the risk of heart failure, arrhythmias, and valvular diseases is also significantly increased in these patients (55). As highlighted in the introduction, multiple mechanisms may be responsible for the increased cardiovascular risk in patients with progressive GFR decline. On the one hand, they include conditions directly related to kidney damage such as chronic anemia, electrolyte and acid-base disturbances, bone metabolism abnormalities, proteinuria, fluid overload, uremia, and arterial stiffening and calcification. On the other hand, traditional cardiovascular risk factors such as hypertension, diabetes, dyslipidemia, obesity, and family history, often present in patients with CKD, are of great importance. To this complex scenario, however, are added multiple so-called emerging factors that may, per se and in addition to the other conditions mentioned above, contribute to cardiovascular events, being also responsible for the residual cardiovascular risk of patients treated with targeted therapies. Among many, these include a pro-inflammatory and pro-thrombotic state, oxidative stress, hyperhomocysteinemia, insulin resistance, endothelial dysfunction (13), and among these, Lp(a) levels appear to be particularly relevant. Currently, however, while the evidence for a causal role of Lp(a) in increasing ASCVD risk in the general population is indisputable, the same conclusion cannot be extended with the same level of evidence to patients with CKD.

Initial studies reported significantly elevated levels of Lp(a) in patients with end-stage disease and hemodialysis (56). We measured plasma Lp(a) in 417 patients with arterial hypertension, 160 of whom had hypertensive nephroangiosclerosis and a creatinine clearance (CrCl) between 30 and 89 mL/min/1.73 m2 (17). In patients with nephroangiosclerosis, plasma Lp(a) levels were significantly higher than in patients without renal involvement, and a highly significant inverse correlation between CrCl and Lp(a) was detected. Furthermore, in these patients, increased Lp(a) was associated with more prevalent coronary artery, cerebrovascular, and peripheral artery disease (17, 21). This observation highlights the fact that the increase in circulating Lp(a) levels occurs from the earliest stages of CKD and may contribute to the presence of ASCVD in these patients. An inverse relationship between CrCl and Lp(a) was confirmed in a further sample of patients with hypertensive kidney disease in whom we measured urinary albumin excretion and characterized apo(a) phenotypes (20). In these patients, urinary albumin losses did not correlate with Lp(a) levels, and the prevalence of different apo(a) phenotypes was comparable across GFR levels. This observation may suggest that kidney damage per se or genes other than LPA might cause increased Lp(a) in patients with mildly reduced CrCl. However, in two studies conducted in subjects with non-hypertensive kidney disease, the inverse relationship between Lp(a) levels and eGFR was observed only among those patients expressing high molecular weight apo(a) isoforms (32, 57).

The first prospective evidence of the relevance of Lp(a) levels on cardiovascular risk in patients with CKD was obtained in the Chronic Renal Insufficiency Cohort (CRIC) study (58). Among the 3744 study participants who were followed for 7.5 years, subjects in the highest quartile of Lp(a) levels (>61.3 mg/dL) had an increased risk of myocardial infarction and mortality (hazard ratio 1.49 and 1.28, respectively). More recently, data from the German Chronic Kidney Disease (GCKD) study were published (22). This study included 5043 subjects with an eGFR between 30 and 60 ml/min/1.73 m2 or an eGFR >60 mL/min/1.73 m2 but with overt proteinuria, who were followed for 6.5 years. In this study, each 10 mg/dL increase in Lp(a) resulted in a significant increase in cardiovascular disease (hazard ratio of 1.065), with the increased risk becoming even more pronounced in subjects with Lp(a) levels >50 mg/dL. Furthermore, subjects with Lp(a) levels >70 mg/dL had their risk increased by more than 30%, regardless of the presence of traditional risk factors. A systematic review of 15 studies including 12260 participants with kidney disease in various stages of evolution, confirmed that high Lp(a) levels were associated with an increased risk of cardiovascular events and death, independently from the presence of traditional risk factors (59). Finally, recently extracted data from the China Renal Data System examined the association of Lp(a) with renal failure and cardiovascular events (60). Among over 50,000 participants who were followed up for 24 and 47 months, a relationship between increasing Lp(a) levels and progression of renal failure was reported along with an association of Lp(a) levels with cardiovascular mortality.

Another aspect that should not be overlooked is the important relationship between Lp(a) levels and the presence of calcific aortic valve disease, which often occurs in association with more advanced stages of CKD. The close relationship between circulating Lp(a) levels and calcific aortic stenosis is well known (61) as is the frequent association between calcific aortic stenosis and CKD (62). Recent data on 6,346 subjects from the MESA (Multi-Ethnic Study of Atherosclerosis) study have demonstrated a significant association between the degree of aortic valve calcification and the incidence of CKD that, however, occurred independent of Lp(a) levels (63). Thus, there is currently no evidence directly correlating the association between aortic valve calcification and CKD to the presence of elevated Lp(a) levels. These and other important aspects related to the role of Lp(a) in CKD have been extensively examined in a recent review by Di Lullo et al., in which the importance of Lp(a) levels for estimation of cardiovascular risk of patients with CKD was reiterated (64).

Taken together, all these findings highlight the importance of Lp(a) as an independent risk factor for ASCVD in patients with CKD, well before they reach the end-stage of disease. Furthermore, because Lp(a) may significantly contribute to residual cardiovascular risk, repeated measurements during CKD progression with the aim of targeting its levels could be a promising strategy to improve cardiovascular outcomes in this high-risk population (65) (**Figure 1**).

**Figure 1:**
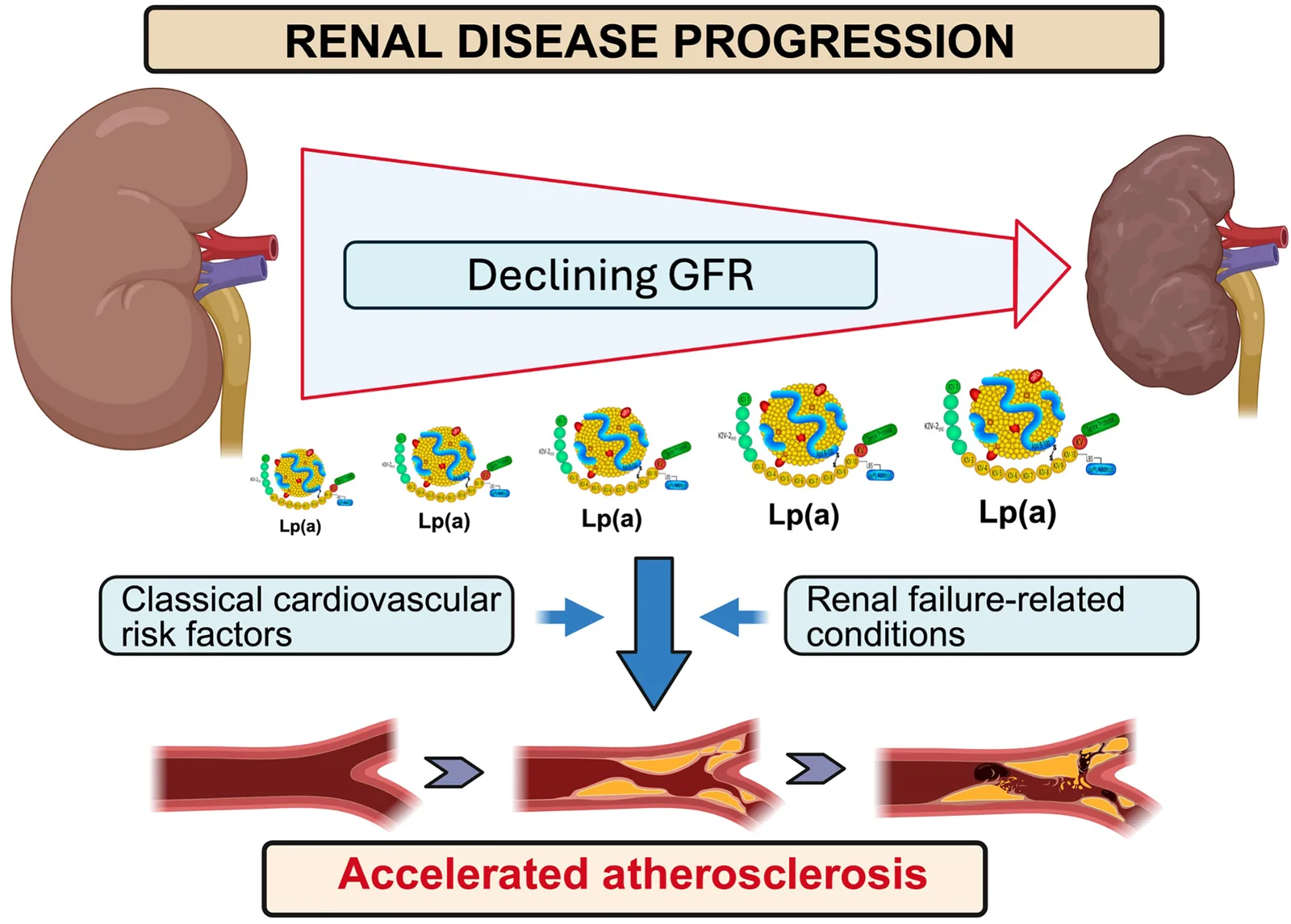
Possible contribution of lipoprotein(a) to accelerated atherosclerosis in chronic kidney disease. Graphic illustration of the possible contribution of lipoprotein(a) to the progression of atherosclerosis in patients with progressive renal failure. Lipoprotein(a) increases progressively with the decline of glomerular filtration rate and, in patients treated according to target parameters for classic cardiovascular risk factors and other specific renal conditions, may contribute to the residual cardiovascular risk of renal patients. Lp(a), lipoprotein(a); GFR, glomerular filtration rate. (created in BioRender. Sechi, L. (2026) https://BioRender.com/h19ommx).

Many studies have also investigated Lp(a) levels in patients with advanced stages of CKD, as well as the influence of dialysis (66). Patients on hemodialysis have been shown to have higher plasma Lp(a) levels compared to healthy subjects (19, 67, 68) and patients with early stage kidney disease (69). In some studies, Lp(a) elevations in hemodialysis patients were limited to those with large apo(a) isoforms (19, 32, 57). Studies in patients with end-stage kidney disease undergoing ambulatory peritoneal dialysis reported significantly elevated Lp(a) levels independent of apo(a) isoform size (57, 67). Although repeated measurements of Lp(a) may be warranted after initiation of dialysis, current evidence does not demonstrate significant reductions in Lp(a) levels with the initiation of either hemodialysis or peritoneal dialysis (70). The observation that dialysis itself does not clear or lower Lp(a) levels may support the notion that increased Lp(a) levels in CKD might be attributed to kidney damage per se and not to differences of the apo(a) phenotypes expressed by patients. Unlike dialysis, renal transplantation decreases significantly Lp(a) levels in patients previously treated with both peritoneal dialysis and hemodialysis (71, 72). Rapid changes of Lp(a) levels after transplantation motivate the need to subsequently perform repeated Lp(a) measurements. Furthermore, Lp(a) changes after transplantation would also be consistent with a leading role of the kidney in Lp(a) removal strongly supporting the hypothesis that Lp(a) elevation in CKD can be ascribed to a loss of functioning kidney tissue. Thus, strong evidence points to the progressive nature of the Lp(a) changes found in patients with CKD, which appear to be the consequence of reduced renal clearance of the lipoprotein rather than increased production. This has also been supported by stable isotope turnover studies (73) and urinary apo(a) clearance studies (74, 75). Increased Lp(a) levels have also been reported in patients with urinary protein wasting and nephrotic syndrome (76, 77), which normalize after remission of the causative condition (78) thus justifying the use of repeated Lp(a) measurement. Even in these subjects, in whom the increase in Lp(a) can occur independently of the eGFR level, the increase in Lp(a) is independent of the apo(a) isoforms (79). However, unlike subjects with non-nephrotic kidney disease, the increase in Lp(a) could be the consequence of increased hepatic synthesis due to urinary protein wasting rather than a reduction in renal catabolism (80). Finally, the observation that Lp(a) levels can be corrected by both renal transplantation and immunosuppressive therapies in nephrotic syndrome may justify the need for repeated Lp(a) measurements in these patients but also in others with progressive kidney disease.

## 5 Interventions to reduce Lp(a)-related cardiovascular risk

Despite the close association between circulating Lp(a) levels and ASCVD, there are currently no widely accessible therapeutic options for its effective reduction. Evidence indicates that most dietary interventions are relatively ineffective on plasma Lp(a) levels (6, 81). The response of Lp(a) to dietary changes is unpredictable, although small reductions were reported when saturated fatty acids were replaced by long-chain carbohydrates and unsaturated fatty acids (82). Furthermore, traditional lipid-lowering drugs have shown minimal and clinically unremarkable effects on circulating Lp(a) (82). Better data, although of modest clinical relevance, were obtained with the two PCSK9 inhibitors evolocumab (27% reduction from baseline) (83) and alirocumab (23% reduction) (84), although the median Lp(a) levels of the patients included in these studies were well below values considered at risk. Therefore, the current consensus for patients with elevated Lp(a) recommends more intensive lifestyle interventions and optimized lipid-lowering treatment in all patients (85).

In parallel with the growing evidence regarding Lp(a) and ASCVD, several new therapeutic approaches have demonstrated the ability to effectively reduce Lp(a) (86, 87). Drug classes with different pharmacodynamic and pharmacokinetic properties are currently under intense investigation (**Table 1**), and additional novel agents are progressing through early development. Lp(a)-targeted treatments act through three main mechanisms and have been tested clinically, all demonstrating substantial efficacy and favorable safety profiles, with some regimens achieving near-complete suppression of circulating levels. However, it is important to emphasize that all studies conducted with these investigational drugs excluded subjects with advanced CKD or on dialysis. Therefore, aspects of these drugs relating to efficacy, dosage, and safety profiles in renal patients cannot be extrapolated and will need to be investigated in appropriately designed clinical trials.

**Table 1:** Investigational drugs for lipoprotein(a) reduction.

Name	Drug class and mechanism	Trial – phase – study population	Duration and Lp(a) reduction	Kidney function	Data in kidney disease	Safety concerns	Ongoing outcome trials
Pelacarsen	ASO	AKCEA-APO(a)-LRx ^89^ Phase 2 completed Patients with ASCVD and Lp(a) ≥60 mg/dL	32 weeks Dose dependent from -35% (20 mg/4 weeks) to -80% (20 mg/week)	Patients with eGFR <60 mL/min/1.73^2^ excluded	No dedicated CKD analysis	Injection-site pain (27%); limits due to size and duration of study; no long-term CKD safety data	Lp(a)HORIZON ^91^ phase 3 (expected 2026) Lp(a)FRONTIERS CAVS phase 2 (expected 2030) ADD-VANTAGE phase 3 (expected 2027)
Olpasiran	siRNA	OCEAN(a)-DOSE ^92^ Phase 2 completed Patients with ASCVD and Lp(a) ≥150 nmol/L	36 weeks Dose dependent from -70.5% to -101% (75-225 mg/12 weeks)	Patients with eGFR <30 mL/min/1.73^2^ excluded	No dedicated CKD analysis	Injection-site pain (17%); limits due to size and duration of study; no long-term CKD safety data	OCEAN(a)^95^ Phase 3 (expected 2026)
Zerlasiran	siRNA	ALPACAR-360 ^93^ Phase 2 completed Patients with ASCVD and Lp(a) ≥125 nmol/L	150 days From -90% (300 mg/16 weeks) to -96% (300 mg/24weeks) and -95% (450 mg/24weeks)	Patients with eGFR <30 mL/min/1.73^2^ excluded	No dedicated CKD analysis	Injection-site pain (7%); limits due to size and duration of study; no long-term CKD safety data	
Lepodisiran	siRNA extended duration	ALPACA ^94^ Phase 2 completed Patients with elevated Lp(a) (median 254 nmo/L)	180 days Dose dependent from -41% (16 mg) to -75% (96 mg) and -94% (400 mg)	Patients with eGFR <30 mL/min/1.73^2^ excluded	No dedicated CKD analysis	Limits due to size and duration of study; no long-term CKD safety data	ACCLAIM-Lp(a)^96^ Phase 3 (expected 2029)
Muvalaplin	Inhibitor interaction apo(a)-apoB	KRAKEN ^97^ Phase 2 completed Patients with ASCVD or equivalent CV risk and Lp(a) ≥175 nmol/L	12 weeks Dose dependent from -48% (10 mg/day) to -81.7% (60 mg/day) and -86% (240 mg/day)	Patients with eGFR <30 mL/min/1.73^2^ excluded	No dedicated CKD analysis	Limits due to size and duration of study; no long-term CKD safety data	

ASO, antisense oligonucleotide; siRNA, small interfering RNA; CKD, chronic kidney disease; CVD, cardiovascular disease; ASCVD, atherosclerotic cardiovascular disease.

Pelacarsen is a single-stranded antisense oligonucleotide (ASO) that, after subcutaneous injection, is released into hepatocytes via the asialoglycoprotein receptor. Once internalized, pelacarsen forms a duplex by binding to LPA mRNA that activates specific RNases leading to selective degradation of the mRNA strand with inhibition of apo(a) production (87, 88). Pelacarsen has been tested in phase 2, randomized, placebo-controlled, double-blind, dose-finding trials in patients with ASCVD and Lp(a) >60 mg/dl (89) and healthy subjects with Lp(a) >35 mg/dL (90). In patients with ASCVD, pelacarsen (20 mg weekly) reduced Lp(a) by 80% at 6 months. In healthy subjects, multiple escalating doses reduced Lp(a) by 59% to 82% after 1 month. To evaluate the possible clinical benefits of Lp(a) lowering, the ongoing phase 3 HORIZON trial (91) recruited patients with ASCVD and Lp(a) >70 mg/dl to evaluate the effects of pelacarsen on the incidence of major cardiovascular events.

Small interfering RNAs (siRNAs) (olpasiran, lepodisiran, zerlasiran) are double-stranded molecules conjugated with N-acetyl-galactosamine that are processed within hepatocytes. Intracellularly, the antisense strand binds to an RNA-induced silencing complex (RISC) that targets LPA mRNA for degradation and concomitant inhibition of apo(a) production (81, 86). When injected subcutaneously, siRNAs reduce plasma Lp(a) in a dose-dependent manner, reaching a reduction from 40% at lower doses to 99% at higher doses (86). In a dose-finding study, olpasiran was administered to patients with ASCVD and Lp(a) >70 mg/dL showing placebo-adjusted reductions of 97% after 8 months with a subcutaneous dose of 75 mg every 12 weeks (91). Comparable and highly significant effects on circulating Lp(a) were reported in subjects with baseline plasma Lp(a) levels >70 mg/dL with zerlasiran (95% reduction after 5 months with a single dose of 450 mg) (92) and lepodisiran (94% reduction after 6 months with a single dose of 400 mg) (93). Similar to pelacarsen, siRNAs are being evaluated in large, long-term studies recruiting patients with established cardiovascular disease to assess the potential clinical benefits of Lp(a) reduction on a composite cardiovascular endpoint including cardiovascular death, myocardial infarction, stroke, and coronary revascularization (94, 95).

In addition to the LPA gene silencing strategies, other agents disrupt the formation of mature Lp(a) particles by interfering with Lp(a) assembly by blocking apo(a)-apoB particle assembly (81, 86). Muvalaplin is an orally active small molecule that selectively targets the kringle 4 domains, disrupting the interaction with apoB. In a 3-month phase 2 study, muvalaplin at a dose of 240 mg/day induced a placebo-adjusted reduction of plasma Lp(a) of 86% (96).

Although still in an investigational phase, there are drugs that are effective in reducing Lp(a) levels and have a good tolerability profile that might be soon approaching clinical use. Whether these very encouraging results will translate into significant reductions in ASCVD remains to be proved, pending the results of ongoing clinical trials. Given the relevance of Lp(a) to cardiovascular risk in patients with kidney disease, the effects of these new treatments may prove particularly beneficial in these patients.

In the meantime, to limit the cardiovascular risk of patients with progressive CKD, it will be necessary to continue to be rigorous in the application of preventive measures that have demonstrated their effectiveness (97). In addition to lifestyle interventions (smoking cessation, weight optimization, salt restriction, exercise as tolerated, avoidance of nephrotoxic substances), optimal blood pressure (target <130/80 mm Hg starting with a renin-angiotensin system inhibitor if not contraindicated) and glycemic control (glycated hemoglobin <7.0%), and use of additional drugs such as SGLT2 (Sodium-glucose cotransporter 2) inhibitors, GLP-1 (Glucagon-like peptide 1) receptor agonists, aldosterone receptor antagonists (spironolactone, finerenone) compatible with residual kidney function, intensive intervention to correct dyslipidemia (LDL <55 mg/dL; triglycerides <150 mg/dL) will be strongly advisable with the use of a statin at an adequate dose, plus possibly ezetimibe and PCSK9 inhibitors.

In high-risk subjects with elevated Lp(a) levels, in addition to strict lipid control, the use of lipoprotein apheresis may currently be considered in selected cases where it is locally available. Lipoprotein apheresis is a highly effective lipid-lowering therapy, but it is semi-invasive, time consuming, and associated with limited patient adherence (98). Previous studies have clearly demonstrated that LDL apheresis reduces cardiovascular events in patients with elevated LDL-C levels (99). Lipoprotein apheresis has also been shown to be effective in reducing cardiovascular risk in patients with concomitantly elevated LDL-C and Lp(a) levels (100). In a retrospective study of 14 patients with ASCVD, elevated Lp(a), and normal LDL-C levels, lipoprotein apheresis reduced Lp(a) levels from a mean of 138 mg/dL to 51 mg/dL (101). A 94% reduction in major cardiovascular events was observed in these patients over a 4-year treatment period. Another retrospective investigation of 25 subjects with Lp(a) >60 mg/dL and normal LDL-C levels, who underwent lipoprotein apheresis for 7.1 years, reported a 67.3% decrease in Lp(a) levels associated with a significant reduction in major cardiac events (102). More recently, an observational study of 170 patients with Lp(a) >60 mg/dL and a diagnosis of ASCVD, in whom other risk factors had been adequately treated, examined the long-term effects of lipoprotein apheresis on cardiovascular outcomes (103). After 12 years of follow-up, a significant reduction in the mean annual event rate was observed compared to a UK Biobank reference cohort of patients with established ASCVD. Thus, current evidence suggests that the prevention of cardiovascular events is a durable effect of lipoprotein apheresis in patients with ASCVD and elevated Lp(a) levels. Among potential mechanisms, the protective effect of lipoprotein apheresis may be linked to the reduced susceptibility of Lp(a) particles to oxidation (104). Experience with lipoprotein apheresis in the context of CKD is limited. However, a retrospective analysis of 9 hemodialysis patients undergoing apheresis over a 4-year period reported a reduction of median Lp(a) levels from 242.7 nmol/L to 64.2 nmol/L, associated with a 57% reduction in the incidence of adverse cardiac and vascular events (105).

## 6 Conclusions

Cardiovascular events are the leading cause of mortality and morbidity in patients with impaired renal function. In these patients, increased cardiovascular risk persists even after adequate correction of traditional risk factors, indicating residual risk, likely related to additional and subtle factors. Among these factors, Lp(a) may play a prominent role, and while traditional lipid-lowering treatments have shown negligible effects on its levels, new effective treatments are emerging into the clinical landscape. A landmark Mendelian randomization analysis reported that genetically predicted, lifelong differences in Lp(a) were associated with an estimated difference in cardiovascular risk (each 10 mg/dl reduction of plasma levels determine a 6% decrease in ASCVD odds), but that this estimate cannot be directly translated into the expected effect of short-term pharmacological lowering (106). Although mendelian-randomization estimates reflect genetically predicted, usually lifelong differences in exposure and cannot automatically be translated into an equivalent short-term pharmacological effect, achieving meaningful reductions of Lp(a) to improve cardiovascular outcomes would require a reduction in levels greater than 50 mg/dl. New therapies reduce circulating Lp(a) up to 90% compared to baseline and thus offer enormous potential for control of residual risk. At present, however, it remains to be seen whether these changes in circulating Lp(a) levels will translate into a proportional reduction in cardiovascular risk. Ongoing trials are testing the hypothesis that a profound reduction in Lp(a) can significantly reduce residual cardiovascular risk. Given the relevance of Lp(a) levels in patients with CKD, demonstration of a meaningful clinical benefit from treatments targeting residual cardiovascular risk could transform Lp(a) from a simple risk marker to a primary therapeutic target.
